# Usefullness of Heparin Calibrated Anti-Xa Activity to Assess Anticoagulant Activity of Apixaban and Rivaroxaban in Emergency Patients Scheduled for Acute Interventions

**DOI:** 10.3390/jcm12216785

**Published:** 2023-10-26

**Authors:** Nada Riahi, Laurence Rozen, Anne Demulder

**Affiliations:** 1 Department of Hematology, Laboratoire Hospitalier Universitaire de Bruxelles LHUB-ULB, Université Libre de Bruxelles ULB, 1020 Brussels, Belgium; nada.riahi@lhub-ulb.be (N.R.); anne.demulder@lhub-ulb.be (A.D.); 2 Laboratory of Hematology, CHU-Brugmann, 1020 Brussels, Belgium

**Keywords:** activity anti-Xa of heparin, correlation between anticoagulant measurements, drug screening, factor Xa inhibitors, laboratory testing

## Abstract

(1) Background: Direct oral anticoagulants (DOACs) require monitoring in some critical clinical situations. The specific tests for DOAC monitoring are not yet available in all labs. The aim of this study was to evaluate if a unique, more widespread heparin-calibrated anti-Xa assay could be suitable to estimate the concentrations of apixaban and rivaroxaban in order to establish an algorithm helping our clinicians in their therapeutic decision for patients treated with DOACs in emergencies. (2) Methods: A first retrospective part allowed us to determine of a conversion factor between the measured DOAC concentration and the deducted anti-Xa heparin activity based on optic density. During the second prospective part, both DOAC concentration (ng/mL) and anti-Xa activity heparin (UI/mL) were measured on the same sample, and the previously determined conversion factor was applied to each UI/mL value. We then compared the calculated and measured DOAC concentration values. (3) Results: The analysis of the derivation cohort confirmed a good correlation, especially between the anti-Xa heparin activity and the apixaban concentrations (r = 0.97). Additionally, we determined heparin-calibrated anti-Xa assay cut-offs for invasive procedures at 0.3 UI/mL and for intravenous thrombolysis at 0.51 UI/mL using ROC curves with a sensitivity at 98% and specificity at 95% for 0.3 UI/mL and a sensitivity at 97.7% and specificity at 88.2% for the cut-off of 0.51 UI/mL. In the validation cohort, we confirmed the agreement between measured and calculated DOAC concentrations for the low values, especially around cut-offs with an excellent negative predictive value for 0.51 UI/mL (94% for apixaban and 100% for rivaroxaban) and a good negative predictive value for 0.3 UI/mL (83.3% for apixaban and 85.7% for rivaroxaban). (4) Conclusions: Our results confirm that it is possible to correctly predict or exclude the presence of apixaban/rivaroxaban in emergency situations when specific tests are not readily available.

## 1. Introduction

Direct oral anticoagulants (DOACs) are recent drugs that act directly and selectively on a specific coagulation factor, either thrombin (for dabigatran) or activated factor X (Xa) (for apixaban, rivaroxaban, and edoxaban). Since they have been on the market for about ten years, their use is constantly increasing, replacing conventional anticoagulants in many indications [1,2]. In randomized control trials, DOACs have demonstrated their non-inferiority compared to warfarin in the prevention of stroke and other thromboembolic complications, particularly in the context of atrial fibrillation. In addition, the incidence of cerebral hemorrhages associated with the use of DOACs is generally lower, although the risk of other types of bleeding may vary between different DOACs, with apixaban showing a comparatively lower risk [3,4]. The fact that they do not require monitoring and the weak interaction with drugs and food make their use more appreciated by clinicians and patients. Another notable advantage is that at least two DOACs (apixaban and rivaroxaban) do not require prior administration of heparin and exert their therapeutic effects within a few hours. Despite their higher direct cost (versus VKAs), the fact that they do not require routine measurements of their anticoagulant intensity reduces their indirect cost and improves compliance. Nonetheless, there are clinical scenarios where it is imperative to determine the concentration of DOACs to ensure appropriate patient management such as recurrence of thrombosis under treatment; urgent invasive procedures such as thrombolysis or surgical interventions; bleeding events; overdose situations; or to ensure therapy levels in patients with multiple factors interfering with pharmacokinetics of DOACs like renal insufficiency, extreme weight, or elderly age. The reference method for measuring DOAC plasma concentration is ultra-performance liquid chromatography tandem mass spectrometry (UPLC-MS/MS) [5]. This sophisticated method is complex and used only in specialized laboratories. Apart from the thrombin time, which can provide information on the concentration of dabigatran, the standard coagulation tests like prothrombin time (PT) and activated partial thrombin time (aPTT) are neither specific nor sensitive to evaluate the plasma concentrations of DOACS [6,7]. Various kits for chromogenic anti-Xa assay can be used with specific controls and calibration curves tailored to each DOAC. Nevertheless, these tests are expensive and not widely available in all institutions. Several studies have highlighted the potential benefits of a single and universal anti-Xa assay that could simplify and make more accessible the estimation of the concentration of F Xa inhibitor in routine laboratories, particularly in emergency situations when specific DOAC assays are not available. This will enable clinicians to make appropriate decisions knowing whether or not the invasive procedure can be carried out under hemostatic safety conditions. In this context, the apixaban and rivaroxaban recommended thresholds to ensure the hemostatic safety are 30 ng/mL for an invasive procedure at high risk of bleeding and 50 ng/mL for IVT thrombolysis in ischemic stroke [8,9,10,11,12].

The aim of our study was to evaluate the use of a unique heparin-calibrated anti-Xa activity assay to assess the DOACs plasma level in patients in emergency situations, particularly when an urgent invasive procedure is essential or IVT is indicated in the context of stroke management. Our goal is also to implement in the future, with the collaboration with our clinicians, a 24/7 decision algorithm that will ultimately make it possible to accelerate patient care.

## 2. Materials and Methods

Our study is divided in two distinct parts. The first part is retrospective, spanning from January 2021 to November 2021, while the second part is prospective, covering the period from December 2021 through May 2023. Our laboratory “LHUB-ULB” is situated across four sites: Porte de Hal, Anderlecht, Schaerbeek, and Horta. The specific DOAC assays are exclusively performed at the Horta site. All samples received from patients treated with either apixaban or rivaroxaban across our four centers of activity were included, regardless of the age or sex of the patients, the indication for treatment, the dose, the frequency of taking the drug, and the time of the last dose. We used residual plasma from patient’s samples to perform the study, in accordance with the approval of the institutional ethics committee for the use of this type of sample. The blood samples were collected in Vacutainer tubes that contained buffered 0.109 M trisodium citrate (1 part of citrate 3.2% in 9 parts of blood). Upon arrival at the lab, they were immediately centrifuged at 1900× *g* for 15 min. The samples were either analyzed immediately, or alternatively, an aliquot of plasma was frozen at −80 °C within 4 h of sampling following the International Council for Standardization in Hematology (ICSH guidelines) [13]. Apixaban or rivaroxaban levels were measured using Biophen Heparin LRT (Hyohen BioMed, Neuville-sur-Oise, France), with ready-to-use liquid reagents. For the specific measurement of apixaban and rivaroxaban, we used specific calibrators depending on the drug used and at a level appropriate to the sample concentration (low or high calibration curves were used). Adequate quality controls were analyzed systematically before each assay. We decide to perform dosages on both CS-5100 and CS-2500 automates (Sysmex—Kobe, Japan) and use different lots of reagents and calibrators to overcome any potential impact of the use of one automate or a single lot of reagent or calibrator. Our center is affiliated with an external quality control program (ECAT, The Netherlands) in accordance with international standard ISO 15189:2022 [14].

### 2.1. Retrospective Part: The Derivation Cohort

During the retrospective part, we collected a complete dataset of measurements of apixaban and rivaroxaban that were performed at the LHUB-ULB Laboratory-site Horta between January 2021 and November 2021. For each significant value of factor Xa inhibitor (≥10 ng/mL), the optical density (OD) data extracted from the specific DOAC assay curve were recorded and then transposed onto the heparin-calibrated anti-Xa assay curve. During the data integration process, the plasma dilutions required by each specific DOAC assay were duly taken into account: a dilution factor of 1/3 was used for the heparin-calibrated anti-Xa assay and the low calibrated apixaban assay, while a dilution factor of 1/20 was applied for the high calibrated apixaban assay. Similarly, a dilution factor of 1/5 was applied for the low-calibration rivaroxaban assay and a dilution factor of 1/10 was used for the high-calibration rivaroxaban assay. Concentration of apixaban or rivaroxaban of each sample expressed in ng/mL was compared to the corresponding value of heparin anti-Xa activity expressed in UI/mL The establishment of a linear relationship between the two variables was necessary for the next step. This enabled us to determine an individual conversion factor (Cfi) for every sample by computing the ratio between the DOAC value and the corresponding anti-Xa activity value. Finally, we determined the average conversion factor (CF) for each anticoagulant (refer to Equation (1)).

Equation (1): Calculation of the conversion factor for each sample, and the mean of all individual calculated conversion samples for each anticoagulant:

Conversion factor for sample 1 CF1 = apixaban 1 (ng/mL)/anti-Xa activity 1 (UI/mL) Conversion factor CF = CF1 + CF2 + CF3..../*n* samples (1)

As an example, for an apixaban dosage at 78 ng/mL, the corresponding OD value extracted was 0.340, which corresponds on the anti-Xa heparin activity curve to 0.8 IU/mL. To calculate the conversion factor, we used the ratio between 78 and 0.80, which corresponded to 97.5 (In this example, we did not use a dilution factor because apixaban low and activity anti-Xa calibrated with heparin have the same dilution: 1/3).

In the next step, we determined the cut-offs of anti-Xa activity expressed in UI/mL and corresponding to cut-offs clinically relevant of 30 ng/mL and 50 ng/mL both for apixaban and rivaroxaban, as mentioned previously. We favored sensitivity over specificity to ensure a maximum of security in the detection of low concentrations of the anticoagulant.

### 2.2. The Prospective Part: The Validation Cohort

During this prospective part of the study, only samples from patients receiving apixaban or rivaroxaban treatments whose drug concentrations were within the lower range were considered. This range is defined as values less than 120 ng/mL for apixaban and less than 100 ng/mL for rivaroxaban. For each sample, we simultaneously measured the concentration of the drug (in ng/mL) using a specific assay and the corresponding anti-Xa activity (in UI/mL) using a heparin-calibrated anti-Xa assay. Using both the mean conversion factor obtained in the first part and the heparin anti-Xa activity prospectively measured, an estimated drug concentration was calculated with Equation (2). Then, we compared the calculated anticoagulant concentration (ng/mL) to the real measurement (ng/mL).

Equation (2): Calculation of anticoagulant by using conversion factor and heparin anti-Xa activity:

Calculated anti-coagulant value = CF × measured heparin anti-Xa activity(2)

As an example, a heparin anti-Xa activity of an apixaban sample of 0.76 IU/mL multiplied by the CF of 97.5 gave an estimated apixaban concentration of 74.1 ng/mL, which we then compared with the real measure of 78 ng/mL.

In this part, we further confirm the cut-off values for heparin anti-Xa activity, which were previously identified and correspond at 30 and 50 ng/mL. We calculated the sensitivity, specificity, positive predictive value, and negative predictive value for each cut-off.

The final step of this study was to propose, after many discussions and in collaboration with our clinicians, an algorithm that could facilitate the management of patients treated with apixaban or rivaroxaban in pre-invasive clinical situations after the simple heparin-calibrated anti-Xa assay.

### 2.3. Statistical Analysis

The Passing Bablok regression test was performed to compare the apixaban or rivaroxaban concentrations and values of heparin anti-Xa activity data on MedCalc version 20.115 Software (Ostend, Belgium). The cut-off levels of the heparin-calibrated anti-Xa assay (expressed in UI/mL and corresponding the recommended cut-offs of 30 and 50 ng/mL) were defined, and the sensitivity and specificity of those cut-offs were determinate using a ROC curve analysis on Graphpad Prism 8 (San Diego, CA, USA). Passing Bablok regression and Bland–Altman plot were used to compare measured and estimated concentrations of apixaban and rivaroxaban on MedCalc version 20.115 Software (Ostend, Belgium).

## 3. Results

In the present study, there were a total of 197 samples from 137 patients. Out of this patient cohort, 100 patients received apixaban, whereas 37 were administered rivaroxaban. The median age of the cohort was 78 years with an equal distribution of gender, comprising 49% women and 51% men.

In the first part (derivation cohort), 126 samples were analyzed, comprising 98 for apixaban (both high and low) and 28 for rivaroxaban (both high and low). We initiated our study by working on two curves for each drug (apixaban high/apixaban low, and rivaroxaban high/rivaroxaban low), but as the study progressed, we decided to focus solely on results obtained with the low anticoagulation calibrated curve since low concentrations are the most important regarding invasive procedures. Indeed, in other non-emergency situations, such as the monitoring of patients with renal failure or extreme weight, specific assays for DOAC measurement can be performed with a longer delay in time. Furthermore, we noted that for higher concentrations of DOACS said to be “in the therapeutic range”, the equivalent in IU/mL very often exceeded 2 UI/mL. With 2 UI/mL being the upper limit of the calibration curve of the anti-Xa assay, equivalency calculations were then more difficult.

**Figure 1:**
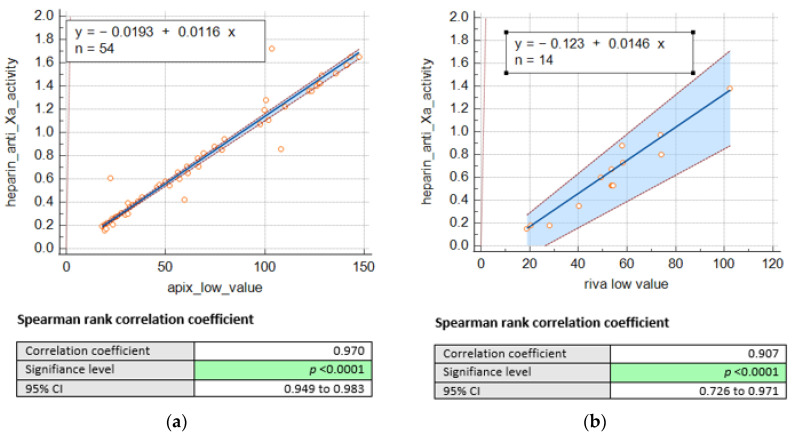
Passing Bablok regression comparing heparin anti-Xa activity (UI/mL) and concentrations of (**a**) apixaban low (ng/mL) and (**b**). rivaroxaban low (ng/mL).

Consequently, we remained with 68 DOACS plasma levels, including 54 for apixaban and 14 for rivaroxaban. Nevertheless, we were able to establish a significant correlation between heparin anti-Xa activities (IU/mL) and drug concentrations (as shown in Figure 1) for apixaban (*n* = 54; r = 0.97, *p* < 0.001) and rivaroxaban (*n* = 14; r = 0.90, *p* < 0.001).

**Figure 2:**
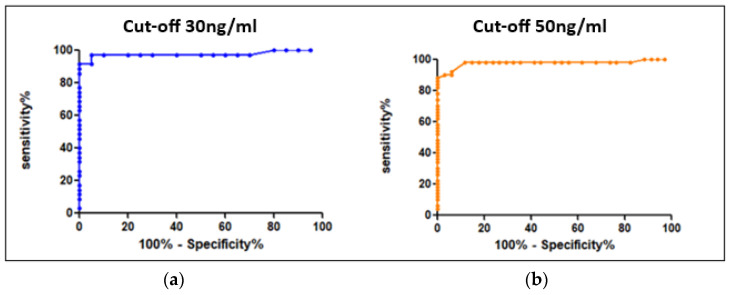
ROC curves for the determination of cut-offs (“hemostatic” security) expressed in heparin anti-Xa activity units (UI/mL) (**a**) for 30 ng/mL and (**b**) for 50 ng/mL.

The values reported above allowed us to determine the conversion factors between heparin anti-Xa activity and specific factor-Xa activity using Equation (1) as previously described. The mean conversion factors were, respectively, 91.5 and 97 for apixaban and rivaroxaban. Using ROC curves (Figure 2), we determined the cut-offs for heparin anti-Xa activity, at 0.3 UI/mL and 0.51 UI/mL, corresponding, respectively, to the recommended thresholds of 30 ng/mL for hemostatic safety and 50 ng/mL for invasive procedures and IVT with a sensitivity of 98% (see Table 1).

In the second phase (validation cohort), the purpose was to validate our initial findings from the first part. We collected only samples with low concentrations, i.e., 71 samples (51 of apixaban and 20 of rivaroxaban). First, we validated the cut-offs of anti-Xa activity values expressed in UI/mL and we calculated the sensitivity, the specificity, the positive predictive value, and the negative predictive value. The cut-off of 0.51 IU/mL highlighted a negative predictive value of 94% for apixaban and 100% for rivaroxaban. The cut-off of 0.3 IU/mL highlighted a negative predictive value of 83.3% for apixaban and 85.7% for rivaroxaban (see Table 2).

**Figure 3:**
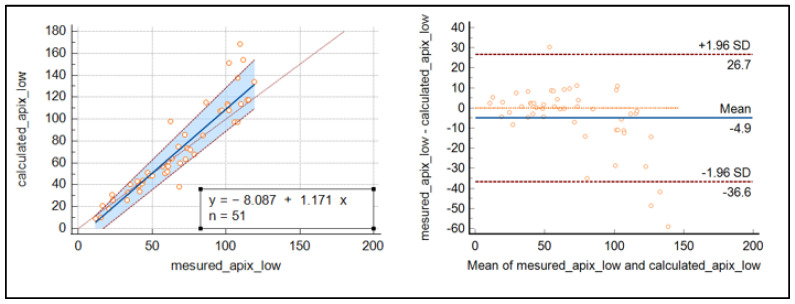
Passing Bablok regression curve and Bland–Altmann plot for the comparison of measured and calculated apixaban.

The Passing Bablok regression and Bland–Altmann plot were used to demonstrate a good relationship between the calculated and measured concentration of apixaban around the cut-off’s values, while for highest concentrations, discrepancies increased as the concentrations were raised (see Figure 3).

**Figure 4:**
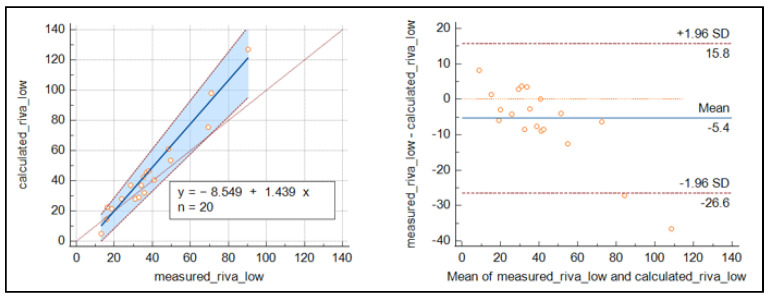
Passing Bablok regression curve and Bland–Altmann plot for the comparison of measured and calculated rivaroxaban.

For rivaroxaban (Figure 4), the number of samples was reduced, and the results were less clear than for apixaban, with slightly higher values around the cut-off for the measured concentrations.

**Figure 5:**
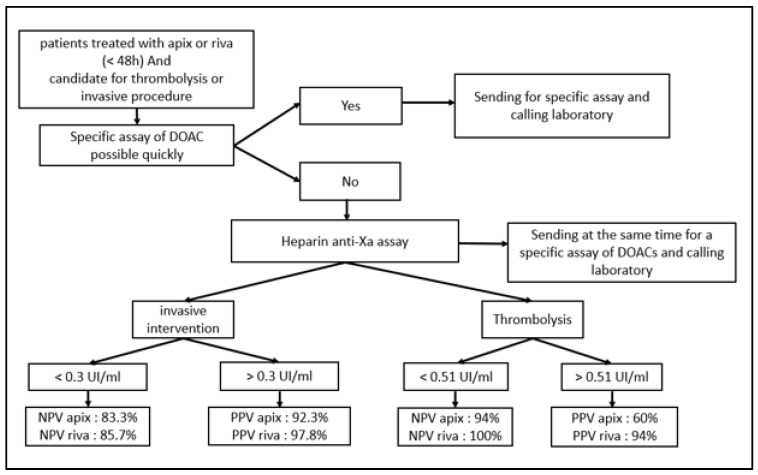
Algorithm of decision in pre-invasive clinical situations for patients treated with apixaban or rivaroxaban.

This good relationship between the heparin calibrated assay and the anticoagulants tested (particularly for apixaban) enabled us to suggest—with the collaboration of our clinicians—a decision algorithm usable 24/7 in emergency situation if specific tests are not readily available (Figure 5).

## 4. Discussion

Our laboratory is multi-site and serves four hospitals each with a Corelab and a clinical emergency department. In addition, two of these hospitals also have a stroke unit. Having a specific dosage for each DOAC on each Corelab is unrealistic and far too expensive. For the moment, the specific dosages are available on one of the sites only, but in the event of an emergency on one of the other sites, the delays in obtaining results are too long. This is why we wanted to offer our clinicians a simple dosing alternative, feasible 24/7. The anti-Xa chromogenic method is similar for all anticoagulants, inhibiting Fxa and the UFH/LMWH calibrant and controls already available on each site. This is why we decided to evaluate this accessible assay to exclude the presence of DOACs above the safety thresholds recommended in the event of an urgent invasive procedure. The most prescribed DOACs in our four institutions are apixaban (maximum safety profile in the elderly) followed by rivaroxaban, which is why we focused on these two drugs. The first retrospective part of our study (derivation cohort) allowed us to show a strong correlation between heparin anti-Xa activity and apixaban concentrations (correlation coefficient r = 0.97 (95% confidence interval (CI), 0.99–0.99) for apixaban low). These findings are also supported by several other studies, such as P. Billoir et al. [15], who demonstrated a significant correlation between heparin anti-Xa activity (UI/mL) and apixaban concentration (ng/mL) (R² = 0.959). Similarly, Boissier, E. et al. [16], who performed a larger study to assess heparin anti-Xa activity in samples from patients treated with apixaban, rivaroxaban, fondaparinux, and danaparoid, also demonstrated a high level of correlation between heparin anti-Xa activity and DOAC concentration (r = 0.99 (95% confidence interval (CI), 0.99–0.99)). A high level of correlation was also demonstrated for apixaban by Maier et al. [17] (R^2^ = 0.96). For rivaroxaban, despite a Spearmann coefficient of 0.90, it is less straightforward to draw firm conclusions due to the smaller size of our study cohort. Nevertheless, it is important to mention that several studies support this correlation [15,16,17]. The heparin anti-Xa activity thresholds we obtained (0.3 and 0.51 IU/mL) are quite consistent with the conclusions of other studies. Similar studies like that of Willekens, G. et al. [18] defined the cut-offs for both apixaban and rivaroxaban as 0.35 IU/mL for 30 ng/mL and 0.58 IU/mL for 50 ng/mL, respectively, whereas Lim, M.S. et al. [19], using different approach by choosing to determine the cut-offs for rivaroxaban and apixaban separately, defined the cut-offs for apixaban at 30 and 50 ng/mL as 0.35 and 0.50 IU/mL, respectively, whereas the cut-offs for rivaroxaban were lower at 0.20 IU/mL for the 30 ng/mL cut-off and 0.35 IU/mL for the 50 ng/mL cut-off. Even lower cut-offs were defined in the study by Maier et al. [17], who reported that heparin anti-Xa activity < 0.16 IU/mL and <0.21 IU/mL reliably excluded a drug level above 30 ng-mL-1 for apixaban and rivaroxaban, respectively. These differences found in the heparin-calibrated anti-Xa activity thresholds is expected and could be explained by the use of different assays and different automates in each study.

In addition, our cut-off values (especially 0.51 IU/mL) are in line with the recommendation of the European Stroke Organization (ESO), which recommended a cut-off value of 0.50 IU/mL for the management of DOAC patients with stroke requiring intravascular thrombolysis [20]. Specifically, seven out of nine ESO participants offer IVT with Alteplase^®^ for patients with acute ischemic stroke according to an anti-Xa activity < 0.5 IU/mL.

Regarding the prospective part (validation cohort), the comparison of the measured and calculated concentrations of apixaban and rivaroxaban was very consistent for apixaban around the recommended cut-off values. The results for rivaroxaban were less conclusive than those for apixaban, as the number of samples was reduced. Our results are in line with those of Boissier, E. [16], who performed her study on a larger cohort of 989 samples and showed good agreement between the estimated and measured levels for each anticoagulant tested.

As part of our study, we proposed to our clinicians an algorithm based on the anti-Xa activity assay to exclude the presence of apixaban and rivaroxaban above the recommended safety thresholds recommended in the event of an urgent invasive procedure. The aim of this algorithm is to facilitate their therapeutic decision making for patients treated with apixaban/rivaroxaban in urgent clinical situations. When used, it should be used with caution and awareness of its limitations. This algorithm must be integrated into the patient’s own clinical situation, taking into account several considerations such as indications for DOAC treatment; concomitant risk factors and diseases, particularly chronic kidney/liver disease and diabetes; laboratory data (Hb, Ht, creatinine, Cockroft–Gault creatinine clearance, eGFR, AST, ALT, etc.); concomitant drugs; and so on. Another prospective clinical study including patient clinical data could prospectively validate the proposed algorithm. The present study has several limitations. By choosing to focus on the lower part of the curve to improve precision up to 100 ng/mL, we reduced the size of our cohort, especially for that treated with rivaroxaban, a drug far less prescribed by our clinicians. Nevertheless, the previously cited studies showing also conclusive results for this drug have encouraged us to present our results, which go in the same direction. In the same vein, edoxaban was not included in our study, as our clinicians rarely prescribe it. Other studies (Boissier et al. [16], Meihandoest et al. [21]) were able to demonstrate equivalence including a wider range of anti-Xa inhibitors. However, their studies cannot be extrapolated to all technical platforms, a restriction applicable in the same way to our own data [22,23].

## 5. Conclusions

In summary and in accordance with other studies, we were able to show a strong relationship between plasma LMWH anti-Xa activity (IU/mL) and apixaban or rivaroxaban concentration in the derivation cohort and in the validation cohort and a relationship between the effective measured concentration and the DOAC concentration predicted by the model. The management of patient treated by DOACS in a clinical emergency situation is challenging. When specific dosages are not available, heparin anti-Xa assay can be a valuable alternative to fairly estimate rivaroxaban and apixaban plasma concentrations. This procedure could be applicable for patients treated in less equipped centers and needing to be transferred to specialized centers for thrombolysis. On arrival, their DOAC status could be already provided.

The use of the proposed algorithm can be the subject of a future prospective study to evaluate the clinical impact.

## Figures and Tables

**Table 1:** Determination of the clinically relevant cut-offs expressed in heparin anti-Xa activity units (UI/mL). AUC = area under the curve.

APIX and RIVA Cut-Offs (ng/mL)	AUC	Sensitivity	Specificity	APIX and RIVA Cutoffs (UI/mL)
30 ng/mL	0.986	98%	95%	0.3
50 ng/mL	0.983	98%	88.2%	0.51

**Table 2:** Sensitivity, specificity, positive predictive value, and negative predictive value for a safe emergency procedure for apixaban and rivaroxaban separately.

DOAC	DOAC	Sensitivity	Specificity	PPV	NPV
Cutoff 0.3 UI/mL	APIX	97.6%	83.3%	97.6%	83.3%
	RIVA	92.3%	85.7%	92.3%	85.7%
Cutoff 0.51 UI/mL	APIX	97%	88.2%	94%	94%
	RIVA	100%	88.2%	60%	100%

## Data Availability

The data presented in this study are available on request from the corresponding author.
